# LEDs driven by AC without transformers or rectifiers

**DOI:** 10.1038/s41598-020-80617-2

**Published:** 2021-01-13

**Authors:** Robin W. Hughes, Mark Warner

**Affiliations:** Isaac Physics, Cavendish Laboratory, University of Cambridge, 19 JJ Thomson Avenue, Cambridge, CB3 0HE United Kingdom

**Keywords:** Applied physics, Electrical and electronic engineering

## Abstract

We explore the driving of LEDs by untransformed AC. An extreme case is driving 1.9 V threshold (red) LEDs with UK mains, peak voltage 325 V. Commonly, driving is by transformed, rectified (DC) supply with a series resistor (where a significant fraction of the power is wasted) to limit current in the LED. With AC, one can instead reactively limit to a maximum current safe for an LED by employing a series capacitive impedance. Cheaper and simpler supplies can thus be employed in some cases. We analyse such non-linear circuits, and also explore questions of duty cycle and power experimentally.

## Introduction

Light emitting diodes, LEDs, ubiquitous in modern electronic devices and lighting, are typically driven by low voltages, rectified to give DC, with a current limiting series resistor, in which a significant fraction of the supplied energy is dissipated. LED lamps, by contrast, have power supplies of considerable sophistication; see a review^1^ and also here in the context of capacitors^2,3^. Such lamps are still miraculously cheap, permitting their mass use from mains power and thereby revolutionising domestic and industrial lighting.

This paper is concerned with an obvious, very low tech alternative to drive LEDs that is never taught to physics and engineering students when they are introduced to these low voltage components. Current limiting can instead be achieved reactively by a single capacitor, even when mains voltage AC is directly applied to individual LEDs, eliminating transformers and rectifiers, and resistive losses in associated resistors. Our simple analysis of, and experiment on, LEDs in non-linear operation shows that they conduct and emit light even in phases when the overall applied voltage is in reverse, and the duty cycle can be extremely high or low, depending on choice of total threshold voltage of (chained) LEDs compared with the driving voltage. The ideas are partially patented^4^ and aspects are employed in a few commercial LED lamps. They are apparently little applied elsewhere, though they could in some applications reduce circuit complexity and cost.

## Capacitive coupling of AC-driven LEDs

**Figure 1:**
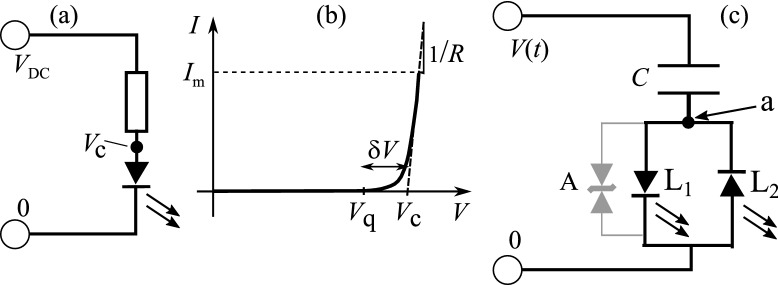
(**a**) A series limiting resistor for a DC-driven LED to prevent excessive current greater than a failure current $I_\text {m}$. (**b**) The *I*–*V* characteristic of an LED is really exponential in the forward direction around $V_\text {c}$, starting at a voltage $V_\text {q}$ say. We approximate *I*(*V*) by an initially very high resistance region followed by a low (differential) resistance *R* region above threshold $V_\text {c}$. (**c**) An anti-parallel pair of LEDs, $\text {L}_1$ and $\text {L}_2$, in series with a capacitor *C*. These two LEDs could also represent two anti-parallel chains of LEDs. Conduction through $\text {L}_1$ or $\text {L}_2$ starts when the voltage at point **a** in the circuit (below the capacitor) is $V_\text {c}$ or $-V_\text {c}$ respectively [light lines: A transient voltage suppressor (TVS) diode (A) in parallel with the LED chains to avoid current surges at first switch-on].

DC-driven LEDs need series resistors to limit current flow because of the extremely steep *I*–*V* variation after their threshold voltage $V_\text {c}$. See Fig. 1a,b. The characteristic in (b), with an exponential switch-on^5,6^, we initially approximate as flat, followed by a steep region (slope 1/*R*) starting abruptly at $V_\text {c}$. We note in experiments where the true exponential switch-on is revealed and discuss the time scales that thereby arise. We return elsewhere to the quantitative analysis of this feature. AC driving is clearly more efficient since power is only developed in the LED. For a supply $V_\text {DC}$, the ratio of the power from the LED to that in the resistor is $\sim V_\text {c} /(V_\text {DC} - V_\text {c} )$ since current, once it flows, is dropped through essentially $V_\text {c}$ in the LED. This ratio of powers is often $\sim 1$, and thus the fraction of power wasted can be significant. By contrast, AC (mains) driving reduces the need for control/power circuitry by using a series capacitor, see Fig. 1c, which offers lossless current limiting, independently of load.

Only capacitors and LEDs, along with elementary notions of reactive impedance, are employed^7^. Simple circuit analysis mostly suffices since the $RC = \tau$ timescale is short compared with the $10^{-2}$ s for the mains. The differential resistance for forward conduction, Fig. 1b, is $R \sim$ 10–30 $\Omega$. Even $C =1\,\mu$F, which will be quite large in what follows, and a differential resistance for an LED of $R = 20\, \Omega$, gives $\tau \sim 2\times 10^{-5}$ s, that is $\sim \times 10^{-3}$ smaller than the mains period. [Our examples are of UK mains at 50 Hz and 230 V RMS, 325 V peak.] We take these simple concepts into a less familiar, non-linear sphere since the diode character of LEDs predominates.

### Capacitive current limiting

Maximum current in the LEDs of Fig. 1c is limited^7^ by the series capacitive impedance $|Z| = 1/\omega C$, where $\omega$ is the angular frequency of the AC supply, represented by $V(t) = V_0 \sin (\omega t)$ where $V_0$ is the peak voltage. Current flows when either LED is conducting, changing the charge on the capacitor and thus the voltage across it. Given that the point **a** of the capacitor is pinned close to either $\pm V_\text {c}$ during conduction, then the current flow is determined by the rate of voltage change of the other end of the capacitor, that is of the supply. Hence the rate of change of charge is $I = C {\text {d}} V/{\text {d}} t$. The maximum current flows when ${\text {d}} V/{\text {d}} t$ is maximal, that is $|{\text {d}} V/{\text {d}} t| = \omega V_0$. Hence, as expected for such a reactive system, $I_\text {max} = C\omega V_0$ for a given supply, and *C* is simply chosen so that $I_\text {max}$ is safe for the LEDs being driven.

### Circuit analysis

Consider the circuit of Fig. 1c, driven by $V(t) = V_0 \sin (\omega t)$. The voltage applied is balanced^7^ by that across the capacitor, *Q*/*C*, plus that at point **a** across the LED(s), $V_\text {a}$:

1 $$\begin{aligned} Q/C + V_\text {a} =&V_0 \sin (\omega t) \;\;\;\; \rightarrow \;\;\;\; I/C +\frac{{\text {d}} I}{{\text {d}} t} {\text {d}} V_\text {a} /{\text {d}} I =&\omega V_0 \cos (\omega t) \end{aligned}$$

where the second form is simply the time derivative of the first. The dependence of $V_\text {a} (I)$ is complicated, with initial and final approximate linearity separated by a shoulder region, a behavior that needs to be considered in forward and reverse condition for the LED pair. The resulting differential equation in *I*(*t*) is thus highly non-linear. The signature of the full form of $V_\text {a} (I)$ emerges in the experimental data below, but the essence of a rich problem, with unexpected results, arises already with the simple model form of *I*(*V*) in Fig. 1b, which ignores the knee details and has $I=0$ for $V<V_\text {c}$ and $I = (V - V_\text {c} )/R$ for $V>V_\text {c}$, with *R* the differential resistance. We return in a future paper to the fully non-linear analysis. We also assume below that the response time of the circuit is fast compared with the driving period $T=2\pi /\omega$.

Further idealising the LEDs, the contribution to the diode voltage from the $V - V_\text {c} = IR$ differential resistive term is taken for the moment to be small; when current flows, it is governed by the impedance of *C*, and the LED voltages $V_\text {a}$ are pinned to $\pm V_\text {c}$. For voltages $|V_\text {a} |<V_\text {c}$, the diodes ensure zero current flow, $I=0$.

Consider a pair of thus idealised diodes (the circuit diagram of Fig. 1c) with a forward conduction voltage $V_\text {c}$: the AC applied voltage *V*(*t*) is dropped across the capacitor as *Q*/*C*, and across the LEDs with a potential dependent upon their state of ON/OFF, with $V_\text {a}$ at point **a**. For switch-on with the capacitor initially uncharged, as *V*(*t*) rises from zero at $t=0$, the point **a**, initially at 0 V, will follow *V*(*t*) upwards as the LEDs are in the OFF state; no charge flows and the lower plate of the capacitor is at the same potential as its upper plate. As $V_\text {a}$ reaches $V_\text {c}$, the LED L$_1$ conducts whilst *V*(*t*) continues to rise, with $V_\text {a}$ now held at $V_\text {c}$ by the action of L_1_. With L_1_ ON, the capacitor charges with a current that varies, but only to a designed maximum. However, at $V(t)=V_0$, the voltage is maximal, ${\text {d}} V/{\text {d}} t=0$. Hence the capacitor no longer charges, and L_1_ goes to the OFF (dark) state. The time is $t = T/4$, a quarter period. The voltage across the capacitor is $\Delta V =V_0 - V_\text {c}$ and remains so until charge can flow off it.

**Figure 2:**
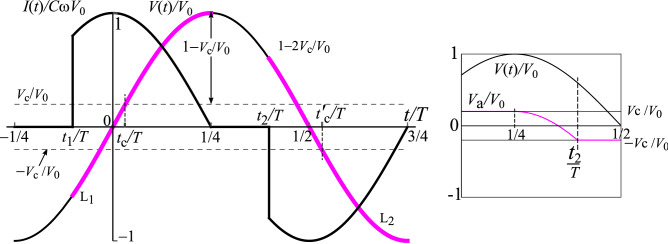
On top of the $V(t)/V_0 = \sin (\omega t)$ AC supply, the periods of emission of the LEDs L$_1$ and L$_2$ are shown (heavy, magenta on-line), starting at times $t_1$ and $t_2$ respectively. The times $t_c$ and $t_c'$ are when the supply crosses the threshold, $V_0\sin (\omega t_c) = \pm V_c$ (light horizontal lines at $\pm V_c$) where conduction would start in simple, resistively-coupled, DC-driven LEDs. Here, conduction starts well before then, indeed for this $V_\text {c} < V_0/2$, at a $t_1 <0$ when the supply is actually in reverse. This example is for $V_\text {c} = 0.2 V_0$, whereupon $t_1 = -0.102 T$. For 50 Hz mains, the off time is $t_1-T/4 = 3.0$ ms. The solid black curves are the current normalised by the maximum current passed by the capacitor, $I/(\omega C V_0)$. Inset: $V(t)/V_0$ and $V_\text {a} (t)/V_0$ against time. While L$_1$ is conducting (until $t= T/4$), the voltage $V_\text {a}$ is pinned to $V_\text {c}$, that is $V_\text {a} = V_\text {c}$. From $t=T/4$ until $t_2$, it drops with the supply as $V_\text {a} = V(t) - \Delta V$ and can be seen to reach $-V_\text {c}$, in this case well before $V(t) = 0$.

With *V*(*t*) now decreasing, the potential of point **a** decreases below $V_\text {c}$: it is fixed to that of the lower capacitor plate which follows *V*(*t*) down since the capacitor is isolated and the potential across it remains fixed. When *V*(*t*) has fallen to $V_0-V_\text {c}$, then $V_\text {a}$ is at 0V and successively when *V*(*t*) drops to $V_0-2V_\text {c}$, then $V_\text {a}$ will drop to $-V_\text {c}$. At this point L_2_ then switches to the ON state. Whilst a current flows through L_2_, this will remain the state, until $V(t) = -V_0$ at which point the capacitor no longer charges, and L_2_ is OFF. The time is $t= 3T/4$. See Fig. 2 where the switch-on time for L$_2$ is marked as $t_2$. The inset shows $V_\text {a} (t)$ constant at $V_\text {c}$ for times after switch-on of L$_1$ but $<T/4$, then dropping between *T*/4 and $t_2$, and then constant at $-V_\text {c}$ from $t_2$ to beyond 3*T*/4.

The time $t_2$, when $V_\text {a} (t)$ reaches $-V_\text {c}$, is determined by

2 $$\begin{aligned} V_0\sin (\omega t_2) = V_0 - 2 V_\text {c} \;\;\;\; \Rightarrow \;\;\;\; t_2 = \frac{T}{2\pi }\sin ^{-1}\left( 1 - 2V_\text {c} /V_0 \right) . \end{aligned}$$

Reverse flow (through L$_2$) starting at the time $t_2$ can occur *before* the supply voltage reverses (at $t = T/2$) if $V_\text {c} < V_0/2$ (case (i)). See Fig. 2 and its inset where $V_\text {c} /V_0 = 0.2$ is chosen as an illustration. For $V_\text {c} > V_0$ (case (ii)), Eq. (2) shows that reverse flow first occurs for $t_2> T/2$, that is when the supply has reversed. In both cases, the on-time extends to 3*T*/4, where $V(t) = -V_0$ is at a minimum.

As *V*(*t*) then rises from $-V_0$ for $t> 3T/4$, point **a** is again isolated and the lower capacitor plate is at a potential $V_0 - V_\text {c}$ higher than the upper plate. The lower plate thus rises as *V*(*t*) does. When *V*(*t*) has risen by $2V_\text {c}$ from $-V_0$, point **a** is at $+V_\text {c}$ and L$_1$ then switches to the ON state. This time is shown as $t_1$ in Fig. 2 and for convenience depicted in the interval $t > -T/4$ in this periodic scheme. The continuing response of L$_1$ is not like its initial switch-on we started with. For instance, it will switch on at a $t_1 < 0$ with $V(t) <0$ for case (i).

**Figure 3:**
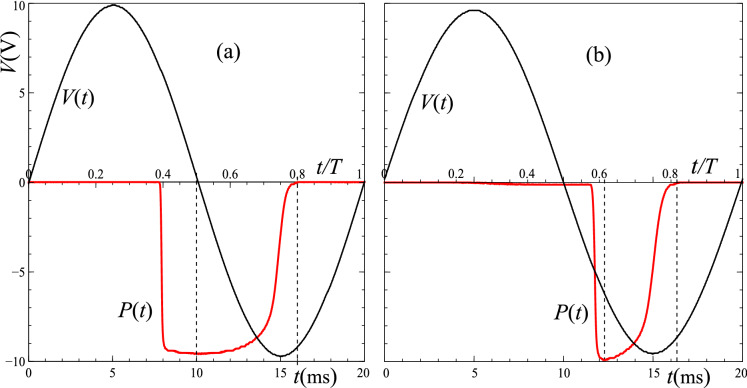
A sinusoidal driving voltage, $V(t) = V_0 \sin (\omega t)$, and the optical power output, *P*(*t*), (inverted for clarity) from one of the two anti-parallel LED chains, L$_2$, for: (**a**) 1 LED in each chain, and $V_0 = 10$ V (case (i)); (**b**) 4 LEDs in each, and $V_0 = 9.5$ V (case (ii)). Vertical dotted lines mark switch off of the optical power, and the points of maximum optical power. The voltage scale refers to *V*(*t*), the optical power being an arbitrary scale for each photo-diode output.

The current, normalised by the magnitude of the maximum current the capacitor can pass, $I(t)/(C\omega V_0)$, is shown as a heavy trace in Fig. 2. The optical power is roughly $I V_\text {c}$ and thus proportional to the current. It is non-monotonic for case (i) where $V_\text {c} < V_0/2$, the maxima being at $t=0$ and $t = T/2$ when $V(t) = 0$ and $|{\text {d}} V/{\text {d}} t|$ is maximal. For case (ii) where $V_\text {c} > V_0/2$, the maxima in brightness are at switch-on, that is at times $t_1$ and $t_2$ of maximum gradient in *V*(*t*) during the interval that conduction is occurring. These regimes are seen graphically in our experiments, see Fig. 3.

### Chains of LEDs, power, duty cycle

The number of LEDs in the LED chains, for a given $V_0$ of the supply, determines whether $V_\text {c} < V_0/2$ or not: $V_\text {c}$ increases linearly with the number of LEDs. The maximum current is purely determined by the capacitor in case (i), $I_\text {max} = C\omega V_0$. In case (ii), $I_\text {max} = C \omega \cos (\omega t_1) = 2C\omega V_0 \sqrt{V_\text {c} /V_0 - (V_\text {c} /V_0)^2}$—as the threshold becomes comparable with $V_0$, the current from the supply reduces (since the LEDs are switched on in increasingly the “right” phase), and the capacitor has to become larger to deliver the same current. In all cases the maximum current passed by the capacitor must be set to be less than the maximum of the LED; $I_\text {max} < I_\text {m}$.

The power delivered is most easily estimated from the charge on the capacitor which varies between $\pm C ( V_0- V_\text {c} )$, all of which is alternatingly dropped through roughly $\pm V_\text {c}$ of the LED chains. Thus during each of $(t_1,T/4)$ or $(t_2, 3T/4)$ an energy of $2 C (V_0 - V_\text {c} ) V_\text {c}$ is delivered. The mean power, *P*, is

3 $$\begin{aligned} P = 4 C V_0 V_\text {c} (1 - V_\text {c} /V_0)/T, \end{aligned}$$

considering that the energy is delivered over a time of *T*/2. The estimate has some approximation since there is an *IR* voltage drop in the LED(s) too on exceeding $V_\text {c}$. See the supplementary material (SM) for examples of anti-parallel chains in LED lamps.

The duty cycle (in effect the fraction of time switched on) for the antiparallel pair(s) is

4 $$\begin{aligned} D = (T/4 - t_1)/ (T/2) = {\textstyle \frac{1}{2}}- 2t_1/T, \end{aligned}$$

with $D \rightarrow 1$ as $t_1 \rightarrow - T/4$ (for $V_\text {c} \ll V_0$).

LEDs are often driven with peak, pulse currents far in excess (perhaps $\times 5$) of their maximal steady current rating. Capacitive driving, because the duty cycle for any individual LED is less than 1/2, and there is a part of the on-time where the emission is below peak, is suited to exploiting over-driving, subject to flicker fusion thresholds (see discussion of flicker below).

To dim a lamp with a given driving capacitance, note that the power simply reduces as $V_0^2$ as $V_0$ is reduced. The lamp continues to emit, so long as $V_0 > V_\text {c}$, the only effect being that the off period gets longer as $V_\text {c} /V_0$ gets larger. No special circuitry is required.

The chains forming the anti-chain pairs do not have to be identical. For instance if some LEDs differ (to possibly change the colour balance), one could have unequal threshold voltages $V_\text {c} ^{(1)}$ and $V_\text {c} ^{(2)}$. Repeating the argument leading to Eq. (2), at $t=T/4$ the voltage across *C* is $\Delta V^{(1) }= V_0 - V_\text {c} ^{(1)}$. Switch on of L$_2$ is when

5 $$\begin{aligned} V(t_2) = \Delta V^{(1)} - V_\text {c} ^{(2)} = V_0 - ( V_\text {c} ^{(1)} + V_\text {c} ^{(2)}) \equiv V_0 - 2\overline{ V_\text {c} } , \end{aligned}$$

where $\overline{V_\text {c} }= {\textstyle \frac{1}{2}}\left( V_\text {c} ^{(1)} + V_\text {c} ^{(2)} \right)$ is the mean threshold voltage for the two chains. At $t=3T/4$ the voltage across *C* is $\Delta V^{(2) }= V_0 - V_\text {c} ^{(2)}$. Switch-on of L$_1$ is when

6 $$\begin{aligned} V(t_1) = \Delta V^{(2)} - V_\text {c} ^{(1)} = 2\overline{V_\text {c} } - V_0 , \end{aligned}$$

so the switch on times $t_1$ and $t_2$ are exactly equivalent, having voltages equally displaced from $\pm V_0$ by $2\overline{V_\text {c} }$. There is no asymmetry in operation, despite the two members of the anti-chain pair differing, allowing scope for substitution of LEDs, e.g. for tuning colour, which is equivalent to tuning the individual $V_\text {c}$s that make up $V_\text {c} ^{(1)}$ and $V_\text {c} ^{(2)}$ . However, there can be problems with reverse bias breakdown in some asymmetric chain pair cases: The reverse bias on chain L$_2$ is $V_\text {c} ^{(1)}$ and, vice versa, that for L$_1$ is $V_\text {c} ^{(2)}$. A standard diode in series will offer reverse bias protection.

#### Robustness of LED assemblies

LEDs, as distinct from conventional, rectifying diodes, are less able to withstand reverse bias. Indeed organic LEDs may have a reverse breakdown threshold, $V_\text {Z}$, comparable in magnitude to their forward conduction threshold. Some typical, white, inorganic LEDs have a reverse breakdown at 5 V with a threshold of 3 V. Each chain in the anti-parallel chain pair is subject to a reverse voltage equal to the forward threshold voltage of the chain it is paired with. Certainly, without additional protection, it is not good policy to have chains of unequal length paired with each other. We have seen, for such cases, (short-circuit) failure of LEDs in chains probably due to this cause. Fluctuation analysis of threshold and breakdown variation shows that LEDs where $V_\text {Z} = V_\text {c}$ exactly are completely unstable as anti-parallel, equal length/character chains. The problem of LED chains of different lengths or different colours can be solved by putting in series with each paired LED chain a standard diode to offer reverse bias protection of a few volts.

Potentially more serious are surges in forward current at switch on: If the assembly is connected to the mains when *V*(*t*) is in the interval $(-V_\text {c} ,V_\text {c} )$, then initially the current flow is zero. However, if for instance *V*(*t*) is outside this voltage range and the capacitor is uncharged, then there would be a large current surge until the capacitor is charged. In fact, the worst case would be switch on when $V(t) = V_0$, the capacitor happens to be reverse charged to $-V_0$, and the LED chain is short, e.g. 1 LED, so that $V_\text {a} \sim 2$V$_0$. In that event, the forward current on attaching to the mains with $V_0 = 325$V would be $I_\text {s} \sim 2V_0/R \sim 650/30 \sim 20$ A, for a time of order $\tau \sim 3\times 10^{-5}$s (that was assumed above; also taking the LED’s differential resistance for *R*). Simple red LEDs we have experimented with seem reasonably robust, but other types are evidently more susceptible to such surges. The LED chains are simply and cheaply protected by being in parallel with a reversed, back to back pair of avalanche diodes, that is connecting points **a** and 0 in Fig. 1c (light lines). In practice this is a single component, a “Transient Voltage Suppressor” (TVS–bi-directional in our case).

### Experiments and closer examination

Applying 325 V AC mains to a 1.9 V LED reversed pair is straight forward, but the difference between $V_\text {c}$ and $V_0$ is so extreme that the off period is very short and harder to illustrate; see “Methods” below. We accordingly used a signal generator with peak voltage in the range $V_0 =$ 0–10.5 V and chain pairs of 1 and of 4 red LEDs, offering $V_\text {c} = 1.9\, \text {V} <V_0/2$ and $V_\text {c} = 7.6 \,\text {V}> V_0/2$ respectively (In series, the LEDs carry the same current, but the voltages add since potential is successively dropped across each chain member. The shoulder voltage is thus multiplied by 4.), corresponding to cases (i) and (ii); see Fig. 3. One can indeed see that the LED switch-on for (i) is before the supply reaches the expected sign, and in (ii) when the supply is of the expected sign, the difference being due to the ratio $V_\text {c} /(V_0/2)$.

In case (i), one sees that peak optical power of L$_2$ is close to $t= T/2$, that is when $V(t) = 0$ and the rate of change of voltage is maximal. One also sees that switch-off is slightly after $t = 3T/4$, the peak of the supply, at variance with the analysis above which was for a sharp cut off at $V_\text {c}$, see Fig. 1b. Rather, we see the effect of the exponential knee extending $\delta V$, say, below $V_\text {c}$, roughly the voltage across the LED chain when the current is low as $V(t) \rightarrow -V_0$ at $t = 3T/4$. After this time, *V*(*t*) and $V_\text {a}$ (which is not quite pinned, as in the simple analysis) decrease by $\delta V$ in an additional time, $\delta t$, given by:

7 $$\begin{aligned} V_0 - \delta V = V_0 \sin [\omega (3T/4 +\delta t)] \;\;\;\;\; \Rightarrow \;\;\;\;\; \delta t = T/(2\pi )\sqrt{2 \delta V/V_0} , \end{aligned}$$

the latter form on expanding the sine for small $\omega \delta t$. Taking $\delta V \sim 0.2$ V, one obtains for the shift of switch off from peak supply of $\delta t = 0.5$ ms, as observed.

Case (ii) follows the same trend—switch on is a little after $V(t) = 0$ for the supply, again due to the *I*–*V* shoulder. This time, peak optical power is close to the switch-on time, but shifted by amounts related to the shoulder. Switch-off is again after the peak in the supply, by a greater amount since the shoulder is $\sim \times 4$ greater than in (i), there being 4 LEDs in series in each chain, rather than 1. The highly non-linear character of the $I-V$ relation makes the full analysis complicated, to which we return elsewhere.

### Mains driving a single, $V_\text {c} =1.9$V anti-parallel LED pair

Consider a maximum current of, say, $I_\text {m} = 20$ mA (giving peak 35 mW output). Given $V_0 = 325$ V and $\omega = 100\pi$ radians.s$^{-1}$, then the required capacitance is $C = I_\text {m} / (\omega V_0) = 196$ nF, and needs to withstand $\Delta V = V_0 - V_\text {c} \sim 325$ V of alternating sign. A ceramic capacitor is required. The off-time of magnitude $t_2 - T/4$ for each of L$_1$ and L$_2$ with $V_\text {c} /V_0 \sim 1/200$ (see Fig. 2) is, from Eq. (2), about 0.5 ms. At first sight phosphors should cover this very short time of no emission, but one has to note that as *t* approaches *T*/4, the emission of L$_1$, and hence the stimulation of phosphors, is getting small, albeit linearly. The power can be estimated from Eq. (3) and gives 23 mW, as compared with the peak of 35 mW; over-driving is easily possible.

#### Flicker

The frequency up to which the human eye sees variation of light intensity depends acutely on the intensity ratio of the temporally varying component to the static background. The faster the variation, the larger this ratio needs to be to perceive intensity variation. It has been found, over about a century of investigation, see for instance the classic works^8,9^, that the eye responds ever more poorly when variation is faster than 50 Hz. Only in extreme cases of the ratio of AC to DC optical power does one perceive flicker at or above 50 Hz. We have found that e.g. red LEDs do not appear to flicker at 50 Hz, while super-brilliant, white (i.e. blue with phosphors) LEDs noticeably flicker in the circuit of Fig. 1c. If connected to a 100 Hz supply (as discussed in SM), there is no perceptible flicker even in extremely brilliant cases.

One can see if LEDs are being driven in the time-dependent way we discuss in this paper by viewing them in the camera of a mobile phone, where there is beating between the 50 Hz and the phone’s frame rate. But it seems that if one perceives residual flicker in a lamp, for instance a fluorescent tube, it is due to other faults and any perceived disturbance is not at 50 or 100 Hz or more. The mobile phone test was a reliable indicator in the 10 types of lamp we took apart (see SM) as to whether an AC or DC drive was being used.

## Discussion and conclusion

We conclude there are advantages to AC driving some LED assemblies capacitively, requiring one, or at most two, driver components and providing quasi continuous optical power, if the LED chain voltage shoulder is small compared with the peak AC voltage. Such methods induce students, and readers generally, to think further about capacitor and diode operation—our approach is very uncommon and seemingly most unfamiliar: Initially counter-intuitive effects are presented, for instance that LEDs can conduct when the supply voltage is still in reverse. Non idealised aspects of LED characteristics, for instance exponential instead of abrupt switch-on, confront the reader immediately our experiments are analysed, and can be understood in terms of finite shoulder regions in the *I*–*V* characteristic.

LEDs are extremely non-linear components and the circuit analysis, though not difficult, requires applying the ideas of reactive circuits carefully. We return to a more fundamental analysis at higher frequencies, that is where 1/*RC* is comparable to driving frequencies.

Because of the high efficiencies of LEDs and the rather small capacitances needed to drive them reactively, capacitive effects already arise from mains wiring. Odd phenomena, such as the “ghosting” of lamps that glow even when isolated from the mains by an open switch, are examined in the supplementary materials (SM). There we also discuss strategies for LED lamps related to the AC drive principles above, though in the literature other directions seem to have been taken, still involving complex circuitry, despite resorting to anti-parallel chain pairs.

## Methods

Rather generic LEDs, red, white and green, were taken to drive capacitively. Typically the maximum current was $I_\text {m} \sim 50$ mA. Output was $\sim 20,000$ mcd at a forward current of $I_\text {f} \sim 30$ mA. A specific red LED was OSHR5111P (TruOpto, from Rapid Electronics). Generally, red LEDs appeared more robust against EOS (electrical overstressing) during switch-on surges and reverse bias when the other (anti) chain was conducting, than were the white and green LEDs.

Ceramic capacitors were used (to withstand reverse bias voltages) and bought from RS. They were in the range 0.1–1 $\mu$F, withstanding 100 - 600 V. A specific example used was a 630 V DC multi-layer Kemet capacitor, RS 9060660. Values were checked with a Fluke 79III multi-meter and found to be $\pm 5\%$ of the nominal value.

For protection of LEDs, a P6KE (Transil) transient voltage surge (TVS) suppressor was adopted. LEDs then suffered no damage, irrespective of how the circuit was switched on.

When not driving the LEDs by the mains directly, signal generators were used, for instance an Agilent 33120A 15 MHz Function / Arbitrary Waveform Generator. Other signal generators from an undergraduate teaching lab were also perfectly adequate. The mains itself, for precision measurements was not suitable since it was highly non-sinusoidal due, presumably, to local loads being applied to it. Indeed the LED non-linearity turned out to be a sensitive tool for examining the character of the mains.

The optical power output was measured from a single LED from the chain pairs of LEDs being investigated. The LED fitted tightly into a very short black plastic tube at one end. At the other end was an equally tightly fitting photo-diode essentially facing head to head with the LED. We employed an Osram BPX65, silicon pin photo-diode.

The driving voltage $V_0 \sin (\omega t)$ and the optical power from the photo-diode voltage output were measured with a PicoScope, USB 2000 & 3000 Series from which the presented signal traces were derived.

To reduce noise, an earthed aluminium foil cover was employed.

## Supplementary Information

Supplementary Information 1.
