# Supplementary material for: LEDs driven by AC without transformers or rectifiers
Source: Sci Rep. 2021 Jan 13;11:963. doi: 10.1038/s41598-020-80617-2 (PMC7807037; doi:10.1038/s41598-020-80617-2)
Supplement: Supplementary file 1 — Supplementary Information 1. [file 41598_2020_80617_MOESM1_ESM.pdf]

Supplementary Material for “LEDs driven by AC without transformers or rectifiers"

Robin W. Hughes[1] **and Mark Warner**[1]

[1]Isaac Physics, Cavendish Laboratory, University of Cambridge, 19 JJ Thomson Avenue, Cambridge CB3 0HE, United Kingdom

ABSTRACT

Illustrations of AC-drive are found in commercial LED lamps, though there is not a direct connection with the principles of the main paper. The effect of mains capacitance is very important for some lamps. Power calculations show significant energy can be wasted by such lamps.

Supplementary Material

“Ghost", commercially-available, domestic LED lamps that seemingly never completely turn off even in open circuit conditions are illustrations of the capacitive effects of the main paper. They give an insight into reactive aspects of mains wiring. See Fig. 1, left and middle. With long cables from switches (in big rooms or when there are multiple switches for a given light), the finite capacitance of mains wiring becomes important. One can calculate the associated quiescent state losses. Such lamps waste much more power in the off state than do incandescent lamps.

Design considerations for mains-driven LEDs and LED lamps

The scheme of a capacitor in series with anti-parallel LED chains allows AC drive without rectification, voltage transformation and current limiting resistors. Concrete examples were given for single LED (chain) pairs. But given that billions of LED lamps are AC-powered from 230V or 110V mains, it is instructive to examine specific example lamps. They appear to fall into two types:
(a) Some lamps are driven by sophisticated, miniaturized power circuitry operating at perhaps 50–100 kHz. Lamp 8, see section “Tear-down", provides its LEDs with 8V DC plus a small 45kHz, almost sinusoidal, component measurable in the light output.
(b) Other lamps are indeed driven by variants of what we have discussed above. Although capacitive methods seem little discussed, the anti-parallel chain pair principle above turned out to be patented, though we did not find it implemented in any of the 10 lamps we dissected. In most cases it is difficult to tell exactly how lamps are driven, but see lamp 7 in section “Tear-down". Miskin and Andersen[1], in their patent, discuss earlier patents that propose AC drive with amplitude matching the

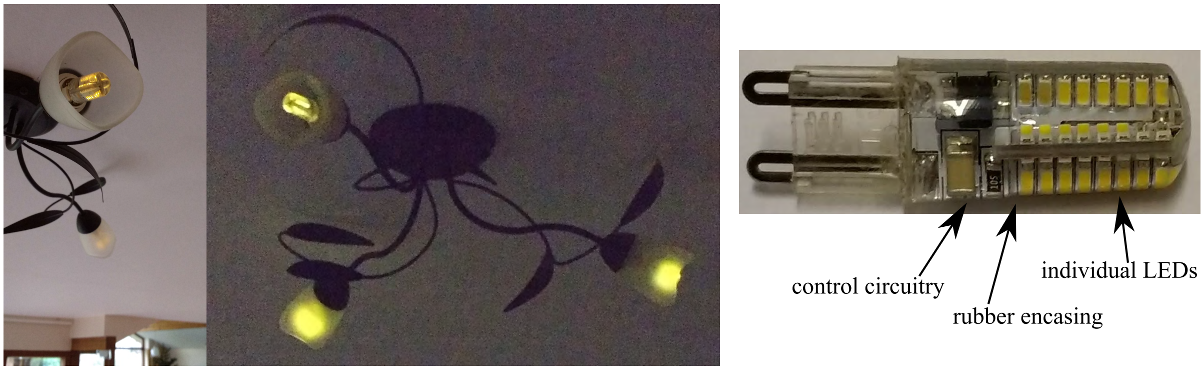

Figure 1. Left: LED lamps glowing (“ghosts") under open-circuit conditions, as seen by day. Middle: as seen by night. Right: The 64-LED ghosting lamp with 4, double-sided vanes each of 8 LEDs. The LEDs and the power/control circuitry are solidly encased in a soft elastomer.

threshold voltage of the LED (chains), which we have seen from Eqn. (M4) (of the main paper) is not a good strategy for power, and from Eqn. (M5) leads to low duty cycles too (since for $V_0 \sim V_c$, the on time $t_1 \to +T/4$) and $D \to 0$. Miskin and Andersen discuss the need for an inverter to produce 12V AC at 20kHz, a high frequency being necessary for miniaturization. As we have seen above, low voltages, and the generation of high frequencies do not seem to be necessary for AC drive. We return below to their suggestion of more complicated LED circuitry.

Anti parallel chains of LEDs for a lamp

For greater output power, as one would want from an LED lamp, one can estimate the required capacitance by re-arranging Eqn. (M4). The series capacitance per watt is:

$$C/P = \frac{T/V_0^2}{4(V_c/V_0)(1-(V_c/V_0))} = 190\text{nF/W} \times \frac{1}{4(V_c/V_0)(1-(V_c/V_0))}. \qquad (1)$$

The final factor depends on V_c/V_0 and is minimal (=1) at $V_c/V_0 = 0.5$. Choice of this ratio affects the length of the off time and also the current. For instance to keep the same length off periods as in the example of Fig. M2 (of the main paper), one would retain $V_c = 0.2V_0$, that is, a chain to give $V_c = 65$V which could be $n = 22$ individual LEDs of threshold e.g. $V_c = 3$V for white light (blue LEDs with suitable phosphors) in each anti-parallel chain of the pair. The numerical factor in Eqn. (1) is $1/(4 \times 0.2 \times 0.8) = 1.6$. The capacitance required would be around 300nF/W. Domestic LED lamp 7 in section "tear-down" below, see Fig. 5, of 5W was revealed to have a 1μF capacitor, a value corresponding to $V_c = V_0/2$.

100hz, capacitive operation of LED chains

Another, related, design strategy is given in figure 2(a). The drive is through ceramic capacitor C_1, is subjected to alternating voltages and is present to limit current flow, and the diodes give current at 100hz. LED lamp 7, see figure 2(b), has the additional C_2 and R_1 components (light lines). The large electrolytic capacitor serves to smooth the current through the LED chain, the series resistor $R_1 = 80\Omega$additionally limits current (perhaps unnecessary?) and two other, large resistors (not shown in the figure) across C_1 and C_2 (for safety). Note that these circuits, in their simplest terms, are quite distinct from a standard,

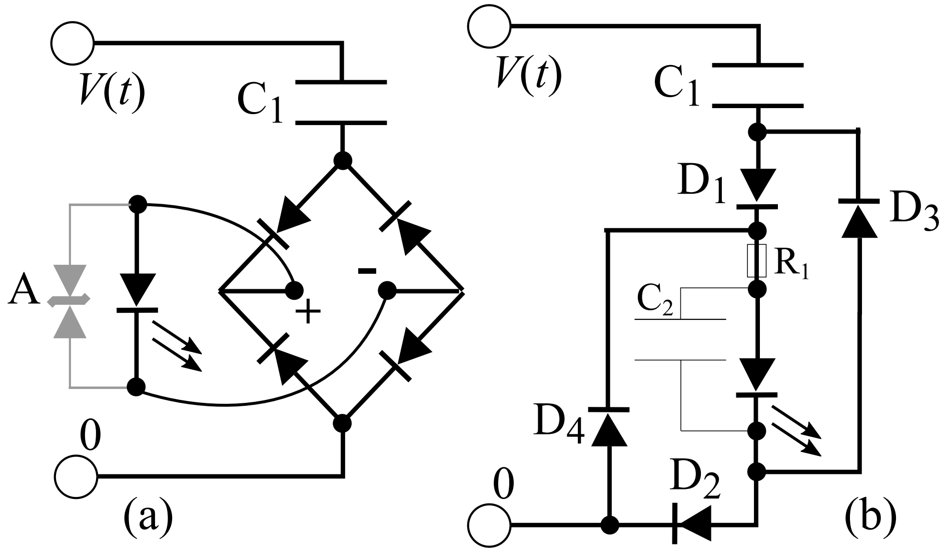

Figure 2. (a) A full-wave rectifier circuit, with current limiting for an LED chain performed by a capacitor (ceramic) on the AC side of the circuit. Current of one sign has a component at 100hz. (b) An equivalent capacitive drive for an LED chain, diodes D_1 – D_4 providing current of one sign. The components (light lines) $C_2 = 4.7\mu$F (electrolytic) and $R_1 = 80\Omega$ are additionally found in LED lamp 7 (see Fig. 5) along with resistors R_2 and R_3, not shown, both 1MΩ, in parallel with C_1 and C_2 respectively.

smoothed, full-wave rectification drive since they are driven via series capacitor C_1, subject to AC, that limits current rather than a resistor, which would be normal with DC. It is also quite distinct from that of a related patent[2], figure 10: the diodes D_1 – D_4 are there replaced by LEDs driving the LEDs (two in parallel representing a chain of LEDs presumably). The problem is that these LEDs associated with the drive (our D_i) are subjected to a reverse bias of magnitude equal to the threshold voltage of the LED chain being driven at 100hz, and would be unlikely to survive that of any LED chain of usable length.

Ghosting lamps

Fig. 3(a) shows a typical wiring of a ceiling lamp with a wall switch. In open circuit, the wires AB and CD form in effect opposite conductors of a capacitance C, of magnitude dependent on the cable length AB/CD. The effective length is still greater if there is more than one switch for the lamp, as in large rooms or in staircases. Then 3-core wire (plus Earth) is required between the ceiling rose and switches, instead of the 2-core wire we have shown. Fig. 3(b) is an equivalent circuit with the series capacitor, but the lamp is more complex than an LED – it works on AC supply whereas a simple LED appearing at this point would light up for part of the first quarter cycle and not again.

Experimental

A three-core cable of length $l = 13.6$m was taken to represent AB/CD of Fig. 3(a) which is a schematic of the strands in the cable going from the lamp ceiling rose to the wall switch. The rose has the live terminal A, the switched live terminal D, the lamp, and the neutral terminal N. The equivalent circuit is shown in Fig. 3(b). The dimensions of the cable in section are shown Fig. 3(c). It consists of a grey plastic sheath surrounding PVC insulated single strand copper wires. This test circuit used the domestic LED lamp of Fig. 1(b). Unwound from its drum, the cable's capacitance was measured[3]. When wound tightly, the capacitance was measured to be approximately 10% higher, an increase that depended on the tightness and which was hard to reproduce. Further small variations occurred on winding and then unwinding. The radii of the wires was 0.62mm in each case. The

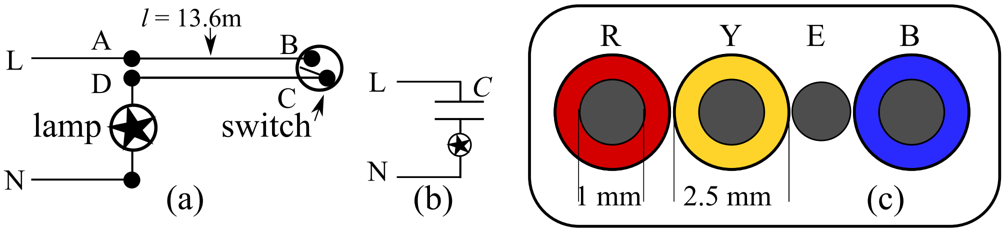

Figure 3. (a) The set up for demonstrating the weakly glowing lamp under open circuit conditions. The conductors AB and CD are taken pairwise from the selection R, Y, E, and B. (b) The equivalent (open) circuit where C is the value of the capacitance between the conductors AB and CD. (c) A 3-core (plus Earth) cable, with the individual wires' (PVC) insulation colour coded R, Y, B. The cores are embedded in a plastic outer. The wire diameters are notionally 1mm, with the Earth a little smaller, and the core-plus-insulation diameters are 2.5mm.

separation of the centres, d, and the various capacitances of the wires taken in pairs was measured. See table 1. The capacitance

Table 1. Dimensions of cable cores, their separation and their mutual capacitances for a 13.6m length (unwound).

pair	nF	d/mm	$1/\ln(d/a)$
R-Y	1.84	2.50	0.721
B-Y	1.52	3.50	0.580
R-B	0.99	6.00	0.442
E-B	2.48	1.75	0.971
E-Y	2.54	1.75	0.971
E-R	1.28	4.25	0.522

per unit length between long, straight, parallel, cylindrical section wires is $C = \pi\varepsilon_r\varepsilon_0/\cosh^{-1}(d/2a) \sim \pi\varepsilon_r\varepsilon_0/\ln(d/a)$, with the latter obtaining for $d \gg a$. The plot of $\ln(d/a)$ against C for the 6 values of the table is very closely linear, indicating consistency in the measurements and that one is comfortably in the large d limit. From the slope, one deduces a relative permittivity reasonable for the insulation plastics used in mains wiring.

It was confirmed that the lamp attached as in Fig. 3(a) indeed dimly lights up in open circuit conditions, never having been previously fully powered up. The dim glow is lost when the L/N supply is disconnected from the open circuit cable. The lamp was also driven in the regular manner by a variable AC supply and was found to light up at an AC peak voltage of about 80V, with a yellowish tinge as in the open circuit case. The wave form of the emitted light in response to 230V RMS was measured using a photo diode and oscilloscope, see Fig. 4, where it is confirmed that there is indeed a threshold for light emission.

Analysis of the LED lamp when ghosting

Domestic LED lamps have complex driving circuitry and a configuration that is not easy to deduce by inspection or indeed by "teardown". However we can crudely estimate the power under open circuit conditions. The effective threshold voltage was observed to be 80V (peak). In each half cycle, current flows into the series capacitor C (the cable to the switch, see Figs. M3(a)

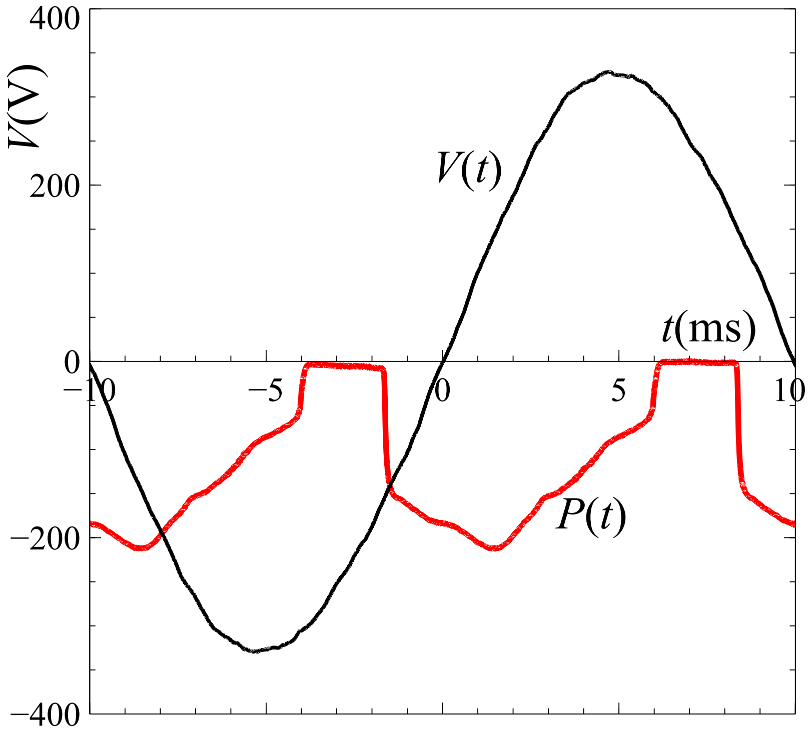

Figure 4. The light output from the LED lamp, $P(t)$ (arbitrary units, shown inverted) investigated for ghosting, along with the driving voltage $V(t) = V_0 \sin(\omega t)$. Note that the duty cycle is quite long, as one would expect for a threshold of ~ 80V compared with $V_0 = 325$V peak, with switch-on before the supply reaches the expected sign. It is also extended by the action of phosphors. See the analysis of driving simple LEDs. During the 10ms repeat (100hz cycle), the lamp is off for 2ms.

and M3(b)) when $V_0 \sin(\omega t) \geq V_c$, storing a charge $Q = C(V_0 - V_c)$, where $V_0 = 325$V is the peak voltage of the 230V RMS UK mains. During charging, an equivalent charge is dropped in series through the V_c of the LED assembly, releasing an energy $U = QV_c = C(V_0 - V_c)V_c$. Using $C \sim 1$nF and V_0 and V_c above, and a repetition rate of 100Hz, the open circuit power is ~ 2.5mW, about $10^{-3}\times$ the on-power. (In fact this power may be an underestimate by at least a factor of two – see the analysis of a simple LED assembly leading to eqn. (M4).) For lamps that are not on for long periods, readers may feel this loss is perhaps an unacceptable effective reduction in efficiency. One must consider that even when the switch cable is shorter, say 1/3rd as long which would be common, the power would still be about one third this amount, but simply be much less visible.

Equivalent incandescent bulbs, say 60W, have a resistance of about $R_{300} \sim 66\Omega$ when cold. One can confirm that $|Z_C| = 1/\omega C \sim 5 \times 10^4 R_{300}$, the magnitude of the impedance of the lead acting as a capacitor, is large compared with R_{300} which can thus be neglected in the overall impedance of the lead plus lamp. The average open-circuit power is thus:

$$\langle P \rangle = \frac{1}{2} \left\langle V_0^2 \right\rangle \frac{R_{300}}{1/(\omega C)} \sim 3.6 \times 10^{-7}\text{W}. \quad (2)$$

The open-circuit power of the equivalent incandescent bulb is tiny, in particular $\sim 10^4$ times smaller than that of the LED lamp.

Apart from the higher wastage of power in the off-state, the glow of LED lamps in bedrooms is unacceptable for sleeping. A solution for multiple, parallel LED lamps is to replace one of the lamps by an incandescent bulb. The charge all to be dropped at V_c is now lost in part through the resistor R_{300} that is in parallel with the LED lamps. This replacement however negates the purpose of using LED lamps since in use the high incandescent power consumption is encountered.

Tear-down of LED lamps

10 lamps were taken apart. The two below (7 & 8) were most illustrative of complex driving circuitry (8) and simple circuitry (7).

Lamp 7

Fig. 5 shows lamp 7 before and after tear down.

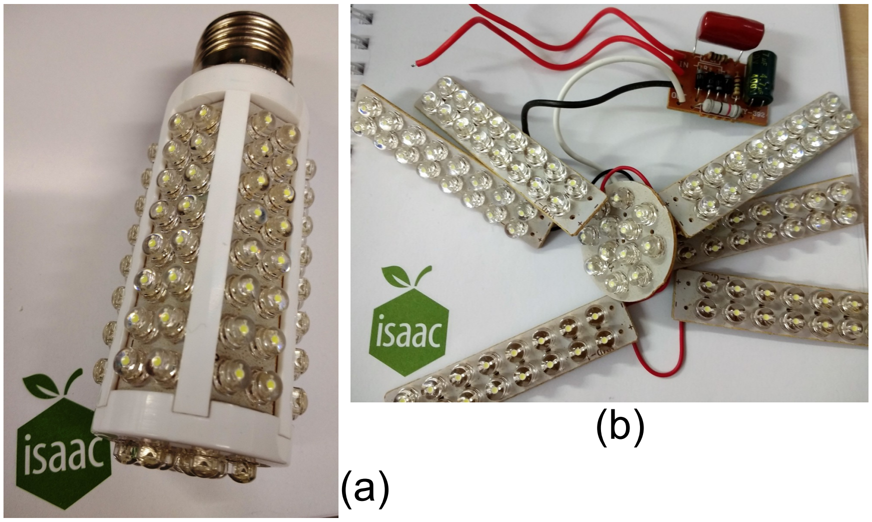

Figure 5. Lamp 7: (a) The lamp before tear down; (b) after tear down.

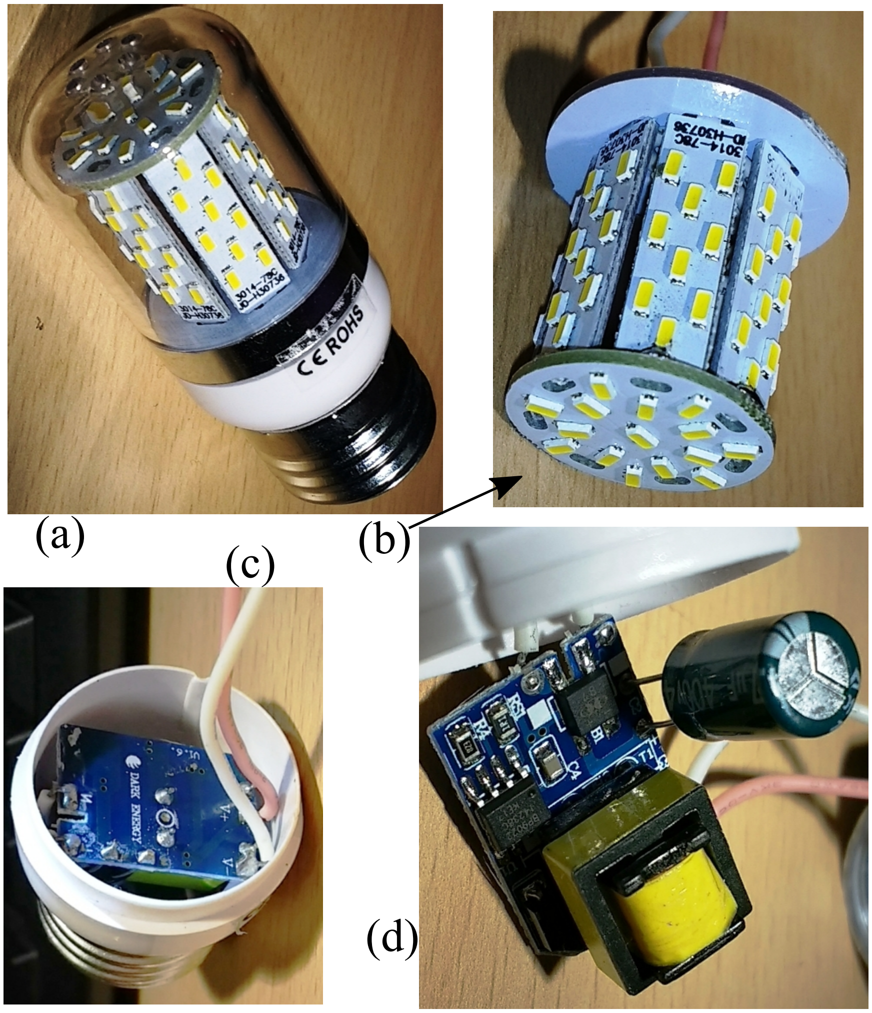

Figure 6. Lamp 8: (a) The lamp before tear down; (b) the LED assembly; (c) the power supply revealed in the screw fitting; (d) the power supply extracted and the capacitor bent out to reveal more underlying circuitry.

Lamp 8

Fig. 6 shows lamp 8 before and during stages of tear down. The lamp consists of 7 panels of 9 LEDs, plus 15 LEDs on the top. They seem to be grouped in threes that are in series with each other (giving a V_c of about 9V, the 26 groups then being in parallel.

References

1. Miskin, M. & Andersen, J. N. AC light emitting diode and AC LED drive methods and apparatus (2004). US Patent: 7489086.

2. Miskin, M. & Huber, C. AC LED lighting element and AC LED lighting system methods and apparatus (2009). US Patent: 2011/0234114 A1.

3. Capacitance meter: “Rapid Electronics" 705a. ±10%.
